# Establishment of Ferroptosis-Related Key Gene Signature and Its Validation in Compression-Induced Intervertebral Disc Degeneration Rats

**DOI:** 10.1155/2023/9020236

**Published:** 2023-02-10

**Authors:** Jiangbo Guo, Yilin Yang, Junjie Niu, Zongping Luo, Qin Shi, Huilin Yang

**Affiliations:** ^1^Department of Orthopaedics, The First Affiliated Hospital of Soochow University, Suzhou, Jiangsu 215000, China; ^2^Orthopaedic Institute, Soochow University, Suzhou, Jiangsu 215000, China

## Abstract

Cell death and functional loss of nucleus pulposus cell play essential roles in intervertebral disc degeneration (IDD). Ferroptosis is a newly identified cell death type, and its role in IDD is still under investigation. Identifying the key genes of ferroptosis in IDD helps to identify the therapeutic targets of IDD. In this study, we downloaded the human IDD mRNA microarray data from the Gene Expression Omnibus and ferroptosis genes from FerrDb, then performed a series of analyses using strict bioinformatics algorithms. In general, we obtained 40 ferroptosis-related differential expression genes (FerrDEGs) and identified six ferroptosis key gene signatures, namely, ATF3, EIF2S1, AR, NQO1, TXNIP, and AKR1C3. In addition, enrichment analysis of the FerrDEGs was conducted, the protein-protein interaction network was constructed, the correlations between ferroptosis key genes and immune infiltrating cells were analyzed, and the lncRNA-miRNA-mRNA ceRNA network was constructed. In particular, ATF3 and EIF2S1 showed the strongest correlation with immune cell function, which might lead to the development of IDD. Finally, the expressions of ferroptosis key genes were verified in the rat compression-induced IDD. In conclusion, this preliminary study analyzed and verified the mechanism of ferroptosis in IDD, laid a foundation for the follow-up study of the mechanism of ferroptosis in IDD, and provided new targets for preventing and delaying IDD.

## 1. Introduction

Low back pain was the most common reason for years lived with disability (YLDs) in 2016, based on the Global Burden of Disease Study [1]. Further, it was among the top 10 causes of YLDs in all 188 countries assessed [1]. Intervertebral disc degeneration (IDD) is an important cause, risk factor, and basic pathological process associated with low back pain, and IDD is a process involving many factors, including oxidative stress, abnormal stress load, aging, trauma, and genetics [2–5]. However, the specific mechanisms and triggering factors of IDD are still debatable. Currently, symptomatic treatment methods are used for managing IDD, such as oral anti-inflammatory analgesics and surgical fusion, rather than interfering with the progress of IDD [6]. However, these treatment methods are alone not adequate to treat IDD, such as recurrent low back pain and degeneration of adjacent spine segments [6]. On account of the heavy social and economic burden caused by low back pain, it is urgent to identify the underlying mechanisms for the onset and progression of IDD and develop new treatment strategies.

Nucleus pulposus is a gel-like structure located in the middle of the intervertebral disc, which synthesizes proteoglycan, absorbs water, provides osmotic pressure, and resists compressive stress [4]. Previous studies have found that apoptosis, autophagy, necroptosis, and pyroptosis are involved in the death and functional loss of nucleus pulposus cells, leading to an imbalance in extracellular matrix metabolism and aggravating the progression of IDD [7–9]. Inhibition of apoptosis, necroptosis, and cell death could partially alleviate the progression of IDD. However, IDD may be a multifactorial pathological process involving multiple cell death modes. Therefore, clarifying other cell death modes would help to understand the mechanism of IDD and develop treatment targets.

Ferroptosis is a new mechanism of cell death, which is different from apoptosis [10]. It is a type of iron-dependent cell death due to the deposition of lipid peroxides on the cell membrane, characterized by lipid peroxidation and free iron-mediated Fenton reaction [10, 11]. The cells with ferroptosis lack the defense system required to eliminate lipid peroxide, accumulating lethal lipid peroxide levels [12]. Studies reported that oxidative stress and iron-dependent reactive oxygen species production primarily contribute to ferroptosis, suggesting that the initiation and effect of ferroptosis require a unique regulatory mechanism [11, 12]. Thus, studying the specific mechanisms of developing and inhibiting ferroptosis would help treat many diseases. Activation of excessive ferroptosis is related to degenerative diseases (e.g., Alzheimer's disease, Parkinson's disease, and acute kidney injury), and impaired ferroptosis often leads to tumor development [10]. Furthermore, some studies have shown that ferroptosis is involved in the IDD progression [13, 14]. Thus, inhibition of ferroptosis may help to treat IDD [15]. However, the research on the mechanism of ferroptosis in IDD is still at the initial stage and needs further exploration. Understanding the role of ferroptosis in IDD might provide therapeutic targets for delaying the IDD progression.

The intervertebral disc has been determined as an immune-privileged organ. The immune privilege is the basis of the intervertebral disc's homeostasis [16]. In addition, studies have shown that, after breaking the physical barrier, immune cell infiltration plays an essential role in the onset and development of IDD [16–18]. However, the relationship between ferroptosis and immune cell infiltration in IDD is still under investigation. Thus, clarifying the correlation between ferroptosis and immune cell infiltration would help understand the IDD progression and provide prevention and treatment targets.

In this study, we found that the gene expression in IDD was enriched in arachidonic acid metabolism according to the Gene Expression Omnibus database dataset and identified the ferroptosis-related differential expression genes (FerrDEGs) in IDD using the FerrDb database. Furthermore, we identified the functions of the genes involved in IDD and the characteristics of ferroptosis key genes and analyzed the correlations between ferroptosis key genes and immune infiltrating cells of IDD. Finally, the expression of ferroptosis key genes and IL-1*β* and TGF-*β*1 was verified in the compression-induced IDD rats. In conclusion, this study identified the key gene signature of ferroptosis in IDD and laid the foundation for future research targeting the prevention and treatment of IDD.

## 2. Material and Methods

**Figure 1:**
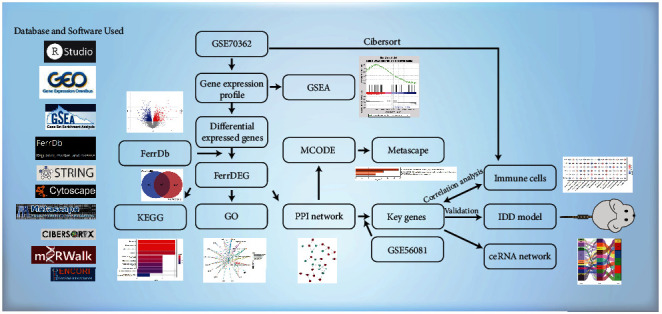
The flow chart of the study. A series of bioinformatics algorithms were conducted to analyze GSE70362. And the compression-induced IDD rat model was constructed to validate the expressions of ferroptosis key genes. The database and software used in this study were presented on the left side of the figure.

Figure 1 shows the flow chart of the database, software, and research methods used in this study.

### 2.1. Dataset Acquisition and Preprocessing

The dataset GSE70362 was obtained from the Gene Expression Omnibus database. This dataset contained mRNA microarray data from 24 intervertebral disc nucleus pulposus samples, of which eight were Thompson grades I and II and were considered the normal control group, and 16 were Thompson grades III-V and were considered the IDD group [19]. The platform was GPL17810. We annotated the probes under the R environment, then got the maximum value of the repeated gene symbol, obtaining the expression matrix.

### 2.2. Gene Set Enrichment Analysis (GSEA)

We downloaded the GSEA software from the official website (https://www.gsea-msigdb.org/gsea/index.jsp) and sorted out expression dataset files and phenotype label files according to the official instructions [20, 21]. We used the GSEA software to conduct the Kyoto Encyclopedia of Genes and Genomes (KEGG) pathway enrichment analysis on the expression profile of GSE70362. The number of permutations was 1000; permutation type was phenotype; the metric for ranking genes was signal2noise, and a pathway with the *p* value < 0.05 was considered a statistically significant enrichment pathway.

### 2.3. Screening of Differential Expression Genes (DEGs) and FerrDEGs

The “limma” package in the R environment was used to analyze the DEGs of the GSE70362 dataset. We selected the *p* value < 0.05 as the screening condition to obtain enough DEGs. We obtained ferroptosis-related genes from the FerrDb database (http://www.zhounan.org/ferrdb), including the ferroptosis marker genes, driver genes, and suppressor genes. The intersections of DEGs and ferroptosis-related genes were obtained using the Venn diagram and were considered FerrDEGs.

### 2.4. Enrichment Analysis of FerrDEGs

We used the “clusterProfiler” package under the R environment to perform Gene Ontology (GO) and KEGG enrichment analysis on FerrDEGs. The GO enrichment analysis included biological process (BP), cellular component (CC), and molecular function (MF), and the adjusted *p* value < 0.05 was considered statistically significant for the enrichment item. *p* value < 0.05 in the KEGG was considered statistically significant for the enrichment item. The top 10 enrichment projects of GO and KEGG were visualized in the histogram under the R environment, and the links between the top 5 enrichment entries of GOBP and FerrDEGs were visualized in the circle diagram.

### 2.5. Construction of FerrDEG Protein-Protein Interaction (PPI) Network and Screening of Ferroptosis Key Genes

We uploaded all FerrDEGs to the STRING version 11.5 website (https://cn.string-db.org/), then constructed a PPI network with confidence > 0.4, and hid the unconnected protein nodes [22]. We used Cytoscape 3.7.2 to visualize the PPI network [23], used the Mcode plug-in to screen the subnet, and used Metascape (http://www.metascape.org) to conduct enrichment analysis of the subnet. The MMC and betweenness algorithms in the plug-in cytoHubba were used to calculate the top 5 proteins, and the union was used to obtain the ferroptosis key candidate genes. The GSE56081 dataset was obtained from the Gene Expression Omnibus database, and the expression of the above candidate genes was extracted for verification under the R environment. Candidate genes with consistent expression trends in GSE70362 and GSE56081 were considered to be ferroptosis key genes.

### 2.6. Correlation Analysis between Immune Infiltration Cells and Ferroptosis Key Genes

The CIBERSORT algorithm under the R environment was used to analyze the immune cell infiltration of IDD. We removed the immune cell types, with an abundance of 0 in all samples. Each immune cell proportion in each sample and each immune cell type contents in all samples were visualized under the R environment. Spearman algorithm was used to analyze the correlations between 16 immune cell types and between 16 immune cells and six ferroptosis key genes. The “ggcorplot” package was used for visualization.

### 2.7. Construction of Potential lncRNA-miRNA-mRNA ceRNA Network of Ferroptosis Key Genes

The miRNA prediction of ferroptosis key genes was carried out using miRWalk 3.0 (http://mirwalk.umm.uni-heidelberg.de/) under the condition of score = 1, and the miRNA validated in miRDB was selected. The miRNA was sorted from large to small pairing numbers, and ENCORI was used (https://starbase.sysu.edu.cn/) to predict the upstream lncRNA of miRNA [24]. We selected the first two miRNAs with upstream lncRNA as candidate potential miRNA, and the lncRNA screening condition was clip data strict ≥ 5. We selected the top two largest of clipExpNum as candidate potential lncRNA. Finally, we used the Sankey map to visualize the lncRNA-miRNA-mRNA ceRNA network of ferroptosis key genes under the R environment.

### 2.8. Construction of a Compression-Induced IDD Rat Model

We constructed a rat compression-induced IDD model as previously described [25]. In brief, ten 12-week-old male Sprague-Dawley (SD) rats were randomly divided into the Sham and IDD groups (*n* = 5 in each group). As previously reported, Co8-Co9 intervertebral discs were compressed with a compression device in the IDD group to establish a long-term compression-induced IDD model in SD rats. However, only Kirschner wires were inserted in Co7-Co10 in the Sham group. The animal protocol was approved by the Institutional Animal Care Committee of the Laboratory Animal at the School of Medicine, Soochow University (Suzhou, China). Rats were raised under standardized conditions, with a light/dark circadian rhythm of 12/12 hours, an appropriate ambient temperature of about 23°C, and free access to food and water. After four weeks, magnetic resonance imaging (MRI) and X-ray imaging of the rat intervertebral disc were performed using a 1.5 T MRI scanner (GE-HDe, USA) and X-ray machine (SHIMADZU, Japan), respectively [26]. The relative quantification of T2 signal intensity was performed using Adobe Photoshop CS6 (California, USA). We calculated the disc height index (DHI) using radiography (X-ray) according to the previous report [27].

### 2.9. Validation of Ferroptosis Key Genes in Compression-Induced IDD Rats

Total RNA in nucleus pulposus was extracted using TRIzol reagent (Beyotime, China), and the RNA concentration was assessed with NanoDrop 2000 (ThermoFisher Scientific, USA). Then, mRNA was reverse transcribed into cDNA using 5× All-in-One RT Mastermix (abm, China), and real-time qPCR was carried out using iTaq Universal SYBR Green Supermix (Bio-Rad, USA) to detect the ferroptosis key genes and IL-1*β* and TGF-*β*1 expression on the CFX96 Real-Time System (Bio-Rad, USA). GAPDH was used as an internal control gene, and the relative expression of the genes was calculated using the comparative Ct method. The forward and reverse primer pairs used for all genes are shown in Supplementary Table 1.

### 2.10. Statistical Analysis

R software 4.2.0 and RStudio 2022.02 (Boston, USA) were used for data analysis and visualization. The comparison of gene expression between two groups was assessed by the two-tailed unpaired *t*-test using GraphPad Prism 8.0 (California, USA). The results were presented as means and standard deviations. Unless otherwise stated, the *p* value < 0.05 was considered statistically significant.

## 3. Results

### 3.1. GSEA

**Figure 2:**
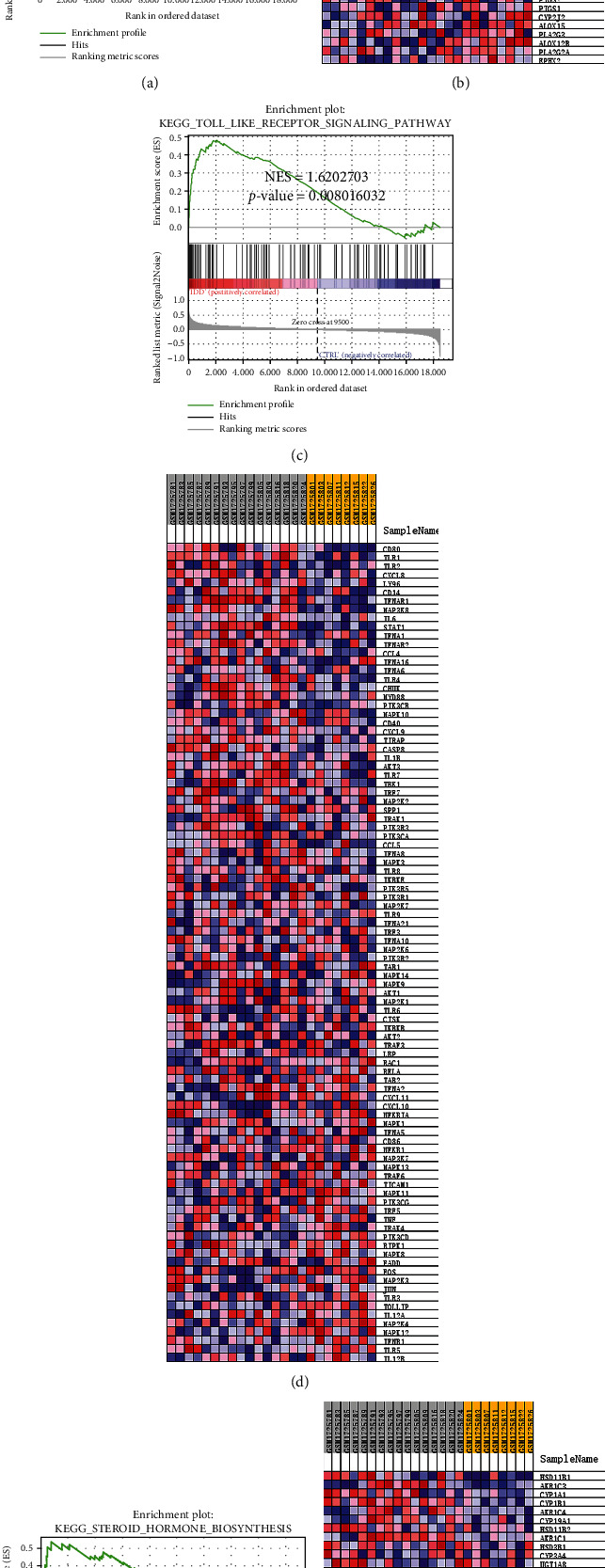
GSEA of the KEGG pathway in IDD. (a, b) Arachidonic acid metabolism in IDD and its related gene expression in each nucleus pulposus sample. (c, d) Toll-like receptor signaling pathway in IDD and its related gene expression in each nucleus pulposus sample. (e, f) Steroid hormone biosynthesis in IDD and its related gene expression in each nucleus pulposus sample.

GSEA demonstrated that the degenerative nucleus pulposus of intervertebral disc was mainly enriched in arachidonic acid metabolism (NES = 1.675538, *p* value < 0.05), Toll-like receptor signaling pathway (NES = 1.6202703, *p* value < 0.05), and steroid hormone biosynthesis (NES = 1.5567781, *p* value < 0.05) (Figures 2(a), 2(c), and 2(e)). The expression of genes related to each pathway in the sample is shown in the heat map (Figures 2(b), 2(d), and 2(f)).

### 3.2. Screening of DEGs and FerrDEGs

**Figure 3:**
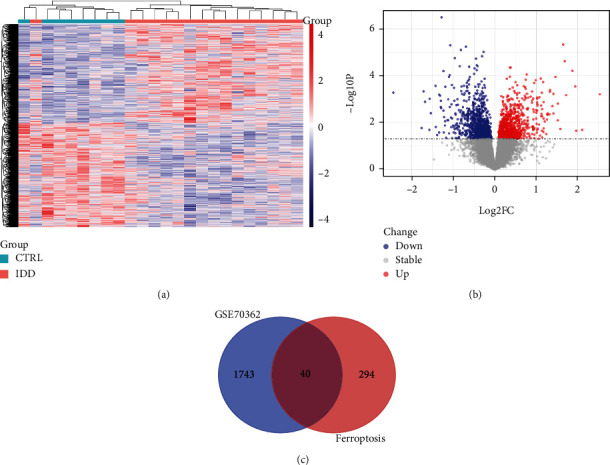
Differential expression analysis and identification of FerrDEGs in IDD. (a) Heatmap of DEGs in IDD. (b) Volcano map of DEGs in IDD. (c) The intersection of DEGs and ferroptosis database genes.

There were 1783 DEGs in total, and the results of differential analyses were visualized using a heat map and volcano map (Figures 3(a) and 3(b)). There were 334 ferroptosis-related genes in total, and the intersection of the two gave 40 FerrDEGs (Figure 3(c)). Refer to Supplementary Table 2 for logFC, *p* value, and regulation of all FerrDEGs. Refer to Supplementary Table 3 for all categories related to FerrDEGs.

### 3.3. FerrDEG Enrichment Analysis

**Figure 4:**
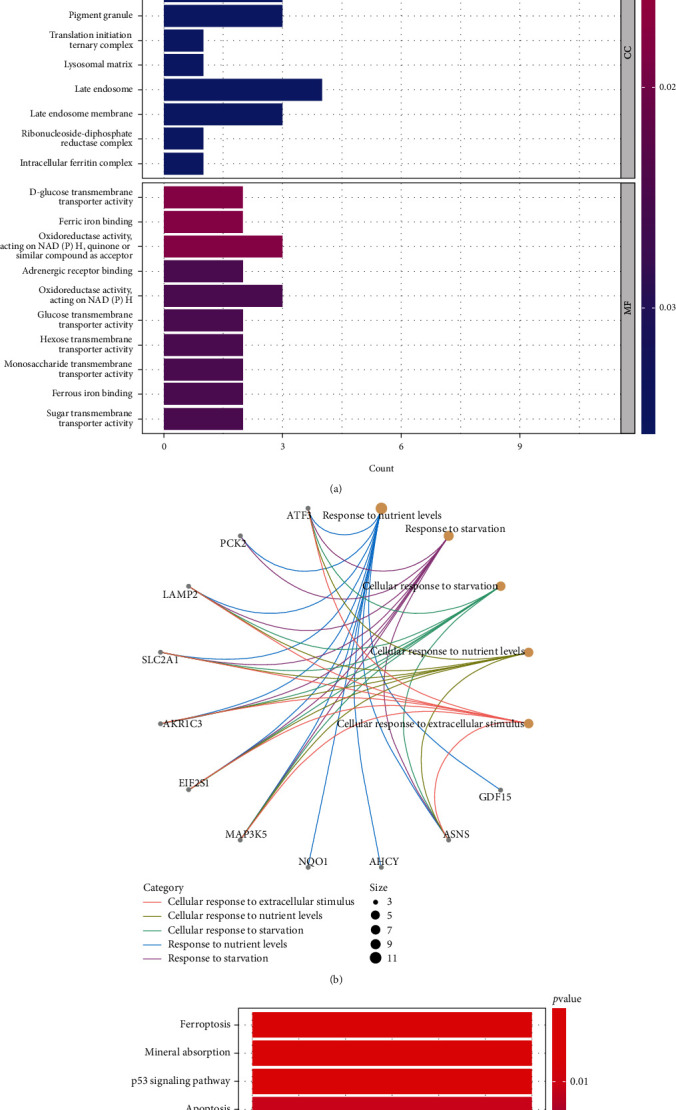
FerrDEG enrichment analysis. (a) GO analysis of FerrDEGs. (b) The top five entries of GOBP and relative genes. (c) KEGG analysis of FerrDEGs.

GOBP enrichment analysis demonstrated that FerrDEGs were primarily involved in the regulation of ferroptosis; GOCC showed that it was enriched in the intracellular ferritin complex. GOMF was primarily involved in ferric and ferrous iron binding; all had adjusted *p* value < 0.05 (Figure 4(a)). ATF3, PCK2, LAMP2, SLC2A1, AKR1C3, EIF2S1, MAP3K5, NQO1, AHCY, ASNS, and GDF15 participated in the first five entries of GOBP enrichment (Figure 4(b)). KEGG enrichment analysis demonstrated that FerrDEGs were primarily involved in ferroptosis, mineral absorption, and the p53 signaling pathway (*p* value < 0.05) (Figure 4(c)).

### 3.4. Construction of PPI Network and Screening of Ferroptosis Key Genes

**Figure 5:**
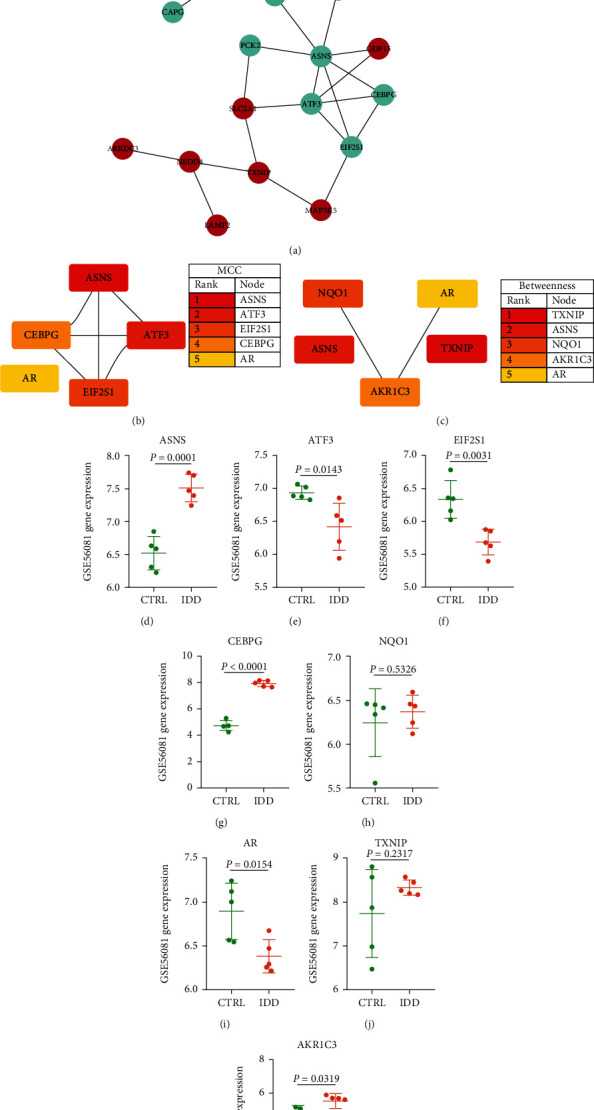
PPI network of FerrDEGs and identification of key genes in FerrDEGs. (a) PPI network of 27 FerrDEGs; red indicates upregulated genes, while green indicates downregulated genes. (b) The MCC algorithm was used to identify the top 5 genes in PPI. (c) The betweenness algorithm was used to identify the top 5 genes in PPI. (d–k) Eight candidate gene expressions in GES56081. Six genes with the same expression trend as GSE70362 were considered key genes of FerrDEGs.

PPI network was generated using STRING and visualized in Cytoscape 3.7.2. PPI network included 27 protein nodes, including 16 upregulated genes and 11 downregulated genes (Figure 5(a)). Mcode plug-in obtained three subnetworks of PPI (Supplementary Figure 1a, b, c). Metascape enrichment analysis showed that the three subnetworks were mainly involved in biological functions, including endoplasmic reticulum stress, negative regulation of cell cycle, and iron overload (Supplementary Figure 1d, e, f). MCC and betweenness calculated the PPI network to identify the top five proteins of their respective algorithms. After combining the results of each algorithm, we identified eight genes, ASNS, ATF3, EIF2S1, CEBPG, NQO1, AR, TXNIP, and AKR1C3 (Figures 5(b) and 5(c)). The expression of eight genes was extracted from the GSE56081 dataset. Unfortunately, the expression of ASNS and CEBPG was not verified. Therefore, the remaining six genes were considered ferroptosis key genes and used for follow-up research objects (Figures 5(d)–5(k)).

### 3.5. Correlation Analysis between Ferroptosis Key Genes and Immune Cell Infiltration in IDD

**Figure 6:**
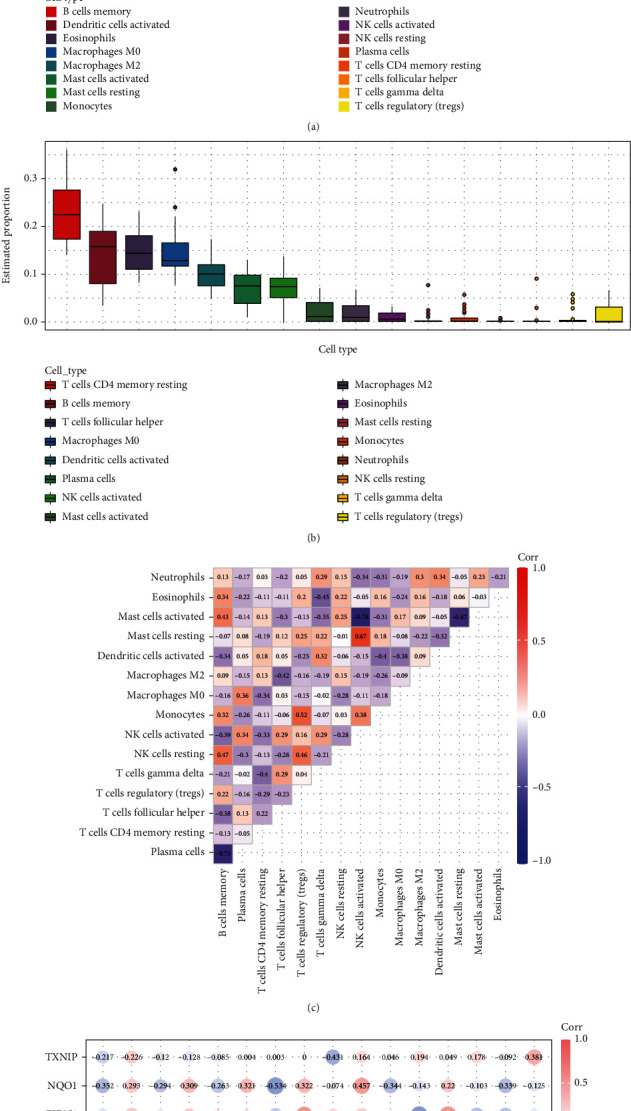
Correlation analyses between ferroptosis key genes and immune cell infiltration in IDD. (a) 16 immune cell type expression in each sample in GSE70362. (b) Each immune cell expression was estimated in all the samples in GSE70362. (c) Correlation analyses between immune cell types. (d) Correlation analyses between immune cell types and ferroptosis key genes.

The CIBERSORT algorithm provided 22 types of immune cell infiltration analyses. In the GSE70362 dataset, we removed the cell types with an expression abundance of 0 in all samples, and 16 types of immune cell types remained. The expression of each immune cell type in each sample is shown in Figure 6(a). Among all immune cell types, CD4 memory resting T cells have the highest expression abundance in intervertebral disc tissue (Figure 6(b)). Spearman correlation analyses of 16 immune cell types (Figure 6(c)) showed that activated NK cells and resting mast cells showed the strongest positive correlation (correlation coefficient = 0.67, *p* value < 0.05), while activated NK cells and activated mast cells showed the strongest negative correlation (correlation coefficient = −0.78, *p* value < 0.05). Spearman correlation analyses of 16 immune cells and six ferroptosis key genes (Figure 6(d)) showed that ATF3 had the strongest positive correlation with M2 macrophages (correlation coefficient = 0.524, *p* value < 0.05), and EIF2S1 showed the strongest negative correlation with activated dendritic cells (correlation coefficient = −0.552, *p* value < 0.05).

### 3.6. Construction of Potential ceRNA Network of Ferroptosis Key Genes

**Figure 7:**
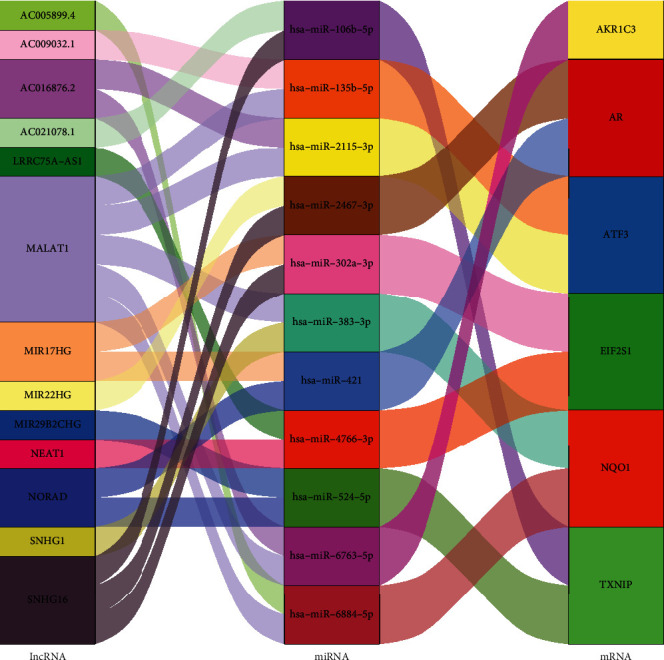
Potential lncRNA-miRNA-mRNA ceRNA network of ferroptosis key genes.

According to our screening conditions, 11 miRNAs and 13 lncRNA were obtained (Figure 7). The 11 potential miRNAs were hsa-miR-106b-5p, hsa-miR-135b-5p, hsa-miR-2115b-3p, hsa-miR-2467-3p, hsa-miR-302a-3p, hsa-miR-383-5p, hsa-miR-421, hsa-miR-4766-3p, hsa-miR-524-5p, hsa-miR-6763-5p, and hsa-miR-6884-5p. AKR1C3 has only one upstream miRNA, and the rest of the genes had two upstream miRNAs. The 13 potential lncRNA were AC005899.4, AC009032.1, AC016876.2, AC021078.1, LRRC75A-AS1, MALAT1, MIR17HG, MIR2HG, MIR29B2CHG, NEAT1, NORAD, SNHG1, and SNHG16. lncRNA MALAT1 showed the most degree of association, followed by lncRNA SNHG16.

### 3.7. Validation of Ferroptosis Key Genes in the Compression-Induced IDD Rats

**Figure 8:**
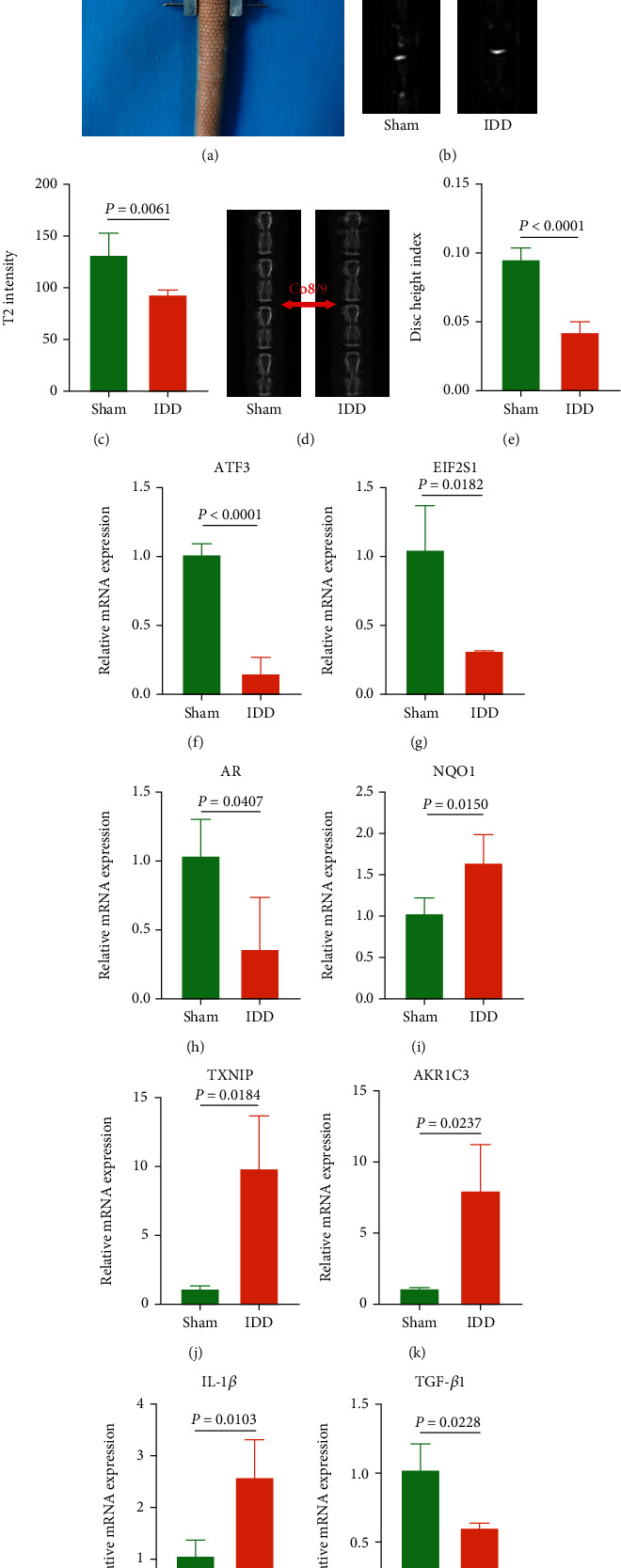
Validation of ferroptosis key genes in compression-induced IDD rats. (a) A compression device was fixed in the rat tail, and Co8-Co9 was compressed. (b) Representative MRI images of the Sham and IDD groups. (c) T2 intensity in the IDD group was significantly lower than that in the Sham group. (d) Representative X-ray images of the Sham and IDD groups. (e) DHI in the IDD group was significantly lower than that in the Sham group. (f–k) Ferroptosis key gene expression in compression-induced IDD rats. (l, m) IL-1*β* and TGF-*β*1 expression in compression-induced IDD rats.

The compression device was successfully fixed on the rat Co7-Co10, in which Co8-Co9 was compressed (Figure 8(a)). After four weeks of compression, MRI and X-ray showed that T2 intensity and DHI in the IDD group were significantly lower than those in the Sham group (*p* value < 0.01; Figures 8(b)–8(e)). The expressions of AR, ATF3, EIF2S1, and mRNA were significantly downregulated in the IDD group, and the expressions of AKR1C3, NQO1, and TXNIP mRNA were significantly upregulated in the IDD group (*p* value < 0.05; Figures 8(f)–8(k)) which were consistent with the results of database analyses. Besides, IL-1*β* mRNA was significantly upregulated in the IDD group compared to the Sham group, while TGF-*β*1 was significantly downregulated in the IDD group compared to the Sham group (*p* value < 0.05; Figures 8(l) and 8(m)).

## 4. Discussion

In this study, we identified six ferroptosis key genes associated with IDD and their biological functions through enrichment analysis, the potential ceRNA network, and the correlation with the immune function in IDD. Six key genes were verified in the rat IDD model, confirming that these genes were vital in the ferroptosis in IDD.

First, we used GSEA to explore the KEGG pathway of gene enrichment in IDD. Arachidonic acid metabolism, Toll-like receptor signaling pathway, and steroid hormone biosynthesis showed the highest enrichment in IDD. GSEA does not rely on differential gene screening conditions but conducts enrichment analysis of the entire gene expression profile [21]. Studies reported that arachidonic acid metabolism and Toll-like receptor signaling pathways were closely related to the onset of ferroptosis [28, 29]. Toll-like receptor-mediated signal promotes inflammatory response in IDD-related metabolic alterations and, thus, plays a role in IDD [30]. Next, we extracted FerrDb database genes and extracted FerrDEGs from the DEGs of GSE70362. These genes were enriched in biological processes and signal pathways related to ferroptosis through GO and KEGG analyses, proving that ferroptosis was involved in the IDD. Finally, we identified six key ferroptosis genes, ATF3, EIF2S1, NQO1, AR, TXNIP, and AKR1C3, through the PPI network and Cytoscape algorithm and the expression verification of GSE56081. And enrichment analysis of PPI subnetworks showed that subnetworks were mainly involved in endoplasmic reticulum stress, cell cycle, apoptotic signaling pathway, and iron overload, indicating these biological processes were important in IDD development.

ATF3, activating transcription factor 3, belongs to the ATF/cyclic AMP response element-binding (ATF/CREB) transcription factor family [31]. ATF3 has a close relationship with the Toll-like receptor signaling pathway, and ATF3 protein could inhibit the expression of many Toll-like receptor-driven proinflammatory genes [32]. Interestingly, as mentioned before, our GSEA showed that the degenerated nucleus pulposus was enriched with the Toll-like receptor signaling pathway. In the study of immune infiltration, we found that ATF3 was positively correlated with M2 macrophages, and as ATF3 level was downregulated in IDD, we speculated that the M2 functional state of intervertebral disc tissue decreased. These findings prove that ATF3, Toll-like receptor signaling pathway, M2 immune response, and ferroptosis play an essential role in IDD. Previous studies reported that ATF3, a wide range of stress sensors, could promote erastin-induced ferroptosis [33]. Meanwhile, several studies have shown that ATF3 knockdown could alleviate the progression of osteoarthritis [34], while some studies have shown that ATF3 is a highly conserved regenerative transcription factor in the vertebrate nervous system [35]. ATF3 protects retinal ganglion cells and promotes the functional preservation of optic nerve after crush [36]. However, only one study showed that ATF3 silencing could inhibit tert-Butyl Hydroperoxide- (TBHP-) induced IDD by inhibiting ferroptosis of nucleus pulposus cells [37]. Further, overexpression of ATF3 inhibits cardiomyocytes' ferroptosis induced by erastin and RSL3 [38]. In addition, ATF3 is a key transcriptional regulator and inhibits inflammatory response [39]. Furthermore, ATF3 can resist LPS-induced inflammatory response [40]. ATF3 could mediate prolonged expression of MMP13 and promote cell proliferation and collagen production in keloid fibroblast cells [41, 42]. Accordingly, the downregulating mechanism of ATF3 expression in IDD may be complex. In IDD, whether the downregulation of ATF3 expression reduces the function of inhibiting inflammatory response, whether ATF3 promotes or inhibits ferroptosis, and the specific effect of ATF3 on extracellular matrix metabolism still need to be further explored.

EIF2S1, eukaryotic translation initiation factor 2 subunit-*α*, is a translation initiation factor [43], and phosphorylation of EIF2S1 is involved in neurodegenerative diseases [44]. EIF2S1 is an endoplasmic reticulum stress marker playing an essential role in maintaining lipid homeostasis [45]. Further, the EIF2S1-ATF4 pathway is closely related to autophagy [46]. EIF2S1 phosphorylation promotes ATF4 activation, increases glutathione synthesis, and improves antioxidant enzyme synthesis, thus, improving the ability of oxygen free radicals [47]. Due to this, the expression of EIF2S1 is downregulated in IDD, and we hypothesized that it might be unable to resist oxidative stress injury, aggravating the IDD progression.

NQO1, NAD (P) H: quinone oxidoreductase 1, plays a controlling role in redox modulation [48]. NQO1 is significantly induced during cell stress, which was verified by our dataset analysis and IDD model [49]. NQO1 has a protective effect on antioxidant stress. Thus, we speculated that the upregulation of NQO1 expression might play a role in inhibiting IDD. Moreover, acacetin alleviates reactive oxygen species produced by TBHP-stimulated nucleus pulposus cells by upregulating the antioxidant protein NQO1 [50]. However, there is little research on the effect of NQO1 in IDD currently, and the biological function of NQO1 still needs further investigation.

AR, androgen receptor, the member of the steroid hormone receptor superfamily, is a class of receptors that function by regulating the transcription of specific genes [51]. Interestingly, our GSEA also showed that IDD was enriched in steroid hormone biosynthesis. AR plays an essential role in many diseases, including complete androgen insensitivity syndrome, spinal bulbar muscular atrophy, prostate cancer, and breast cancer [52]. Several studies have shown that estrogen and its receptor could play a protective role in IDD [53, 54]. However, the relationship between AR and IDD has not been reported yet. An animal study investigating temporomandibular joint osteoarthritis reported that excessive mechanical stress stimulation was related to severe articular cartilage degeneration in the estrogen and androgen deficiency group [55]. Consistent with this work, AR was downregulated in our dataset analysis and compression-induced IDD model; we could speculate that androgen and AR might play a protective role in IDD, and the reduced expression of AR in IDD might lose its protective function and promote the progression of IDD.

AKR1C3, human Aldo-keto reductase family 1 member C3, is a hormone activity regulator [56]. On the one hand, AKR1C3 produces effective androgens in peripheral tissues and could activate AR [57]. Therefore, AKR1C3 might activate AR to protect the intervertebral disc from degeneration. And, the previous report has demonstrated that HOXB4 could serve as a transcriptional activator for AKR1C3 and suppress the ferroptosis of the H9C2 cells [58]. This suggests that the upregulation of AKR1C3 might inhibit ferroptosis in IDD somehow. On the other hand, AKR1C3 and *β*-catenin signaling may have a synergistic effect, and *β*-catenin signaling is often upregulated and contributes to IDD, suggesting AKR1C3 could promote IDD development through the *β*-catenin pathway [59, 60]. It seems that AKR1C3 cuts both ways in IDD. Therefore, we will design a thorough biological functional experiment to explore the mechanism of AKR1C3 in IDD.

TXNIP, thioredoxin interacting protein, is associated with neurodegenerative diseases [61]. TXNIP could promote nucleus pulposus cell pyroptosis, while TXNIP inhibitor Morin could alleviate nucleus pulposus cell pyroptosis [62]. TXNIP promotes oxidative stress by inhibiting the thioredoxin (TRX) system, and studies have shown that its expression is upregulated in brain diseases such as stroke [63]. In our study, TXNIP was upregulated in IDD in both databases and IDD models. Therefore, we could speculate that TXNIP may not only induce pyroptosis but also play an essential biological function in elevating ferroptosis through promoting oxidative stress in IDD.

Immune infiltration analysis is often used to analyze the distribution of immune cells in the microenvironment of tumors and other diseases [64]. We analyzed the immune infiltration of IDD and found that 16 types of immune cells were distributed in the intervertebral disc; not all immune cell types are expressed in the intervertebral disc, which might be determined by its own tissue characteristics. The intervertebral disc is a tissue with no blood supply and lymphatics [65]. The unique structures and molecular factors expressed in the intervertebral disc show inhibitory effects on immune cells and cytokine infiltration [16]. However, according to the correlation analysis of 16 immune cells, activated NK cells showed positive and negative correlations with resting and activated mast cells, respectively. Therefore, the function of NK cells is closely related to mast cells in intervertebral disc tissue. Furthermore, the correlation analysis between ferroptosis key genes and immune cells showed that ATF3 had the strongest positive correlation with M2 macrophages. EIF2S1 and activated dendritic cells showed the strongest negative correlation. Meanwhile, the expression of ATF3 and EIF2S1 was decreased in IDD, suggesting increased inflammation and reduced repair capability in IDD. Previous studies reported that ATF3 is a key transcriptional regulator that inhibits inflammatory response [39]. The ATF3 overexpression promotes macrophage migration and M2 phenotype, while the ATF3 knockdown leads to the opposite effect, which is consistent with our hypothesis [39]. Therefore, we investigated the IL-1*β* and TGF-*β*1 expression in the IDD model, and we found that IL-1*β* was significantly upregulated while TGF-*β*1 was significantly downregulated in IDD. We verified IL-1*β* and TGF-*β*1 expression in the IDD model and confirmed the above hypothesis.

ceRNA mechanism plays an essential role in treating many diseases, including IDD [66]. Using an online database, we could predict mRNA and its upstream miRNA and lncRNA. Among lncRNA, lncRNA MALAT1 showed the strongest association, followed by lncRNA SNHG16. Therefore, these genes might be involved in regulating ferroptosis genes in IDD. A previous study showed that lncRNA MALT1 could suppress inflammation, inhibit nucleus pulposus cell apoptosis, promote cell proliferation, and attenuate aggrecan degradation [67]. Knockdown of lncRNA SNHG16 suppressed cell viability and induced apoptosis in chondrocytes [68]. Yet, there is no evidence about the biological function of lncRNA SNHG16 in IDD. However, this is a preliminary prediction and needs further experimental verification.

The limitation of the study was that we only verified the expression changes of ferroptosis key genes in IDD, and further experiments are needed to study their biological functions in IDD. In addition, the sample size of the dataset used in this study was small due to the lack of research on IDD in a public database. Meanwhile, obtaining normal human intervertebral disc tissue (control) is difficult and limits the progression of related research. Despite this, the animal model may cause inevitable bias as they have a different structure from the human sample. However, the compressive loading IDD model could lead to cell death and impaired matrix synthesis, and the IDD model has the advantage of controllability [69]. Therefore, we verified ferroptosis key genes in the compression-induced IDD rats. Besides, MRI and X-ray imaging confirmed that we successfully constructed the IDD model. In the follow-up study, complete biological function experiments are needed to investigate the role of these genes in IDD.

## 5. Conclusion

This study identified six ferroptosis key genes in IDD, their biological processes involved, and the correlation between immune infiltration cells, providing a reference for follow-up studies investigating the mechanism and treatment of IDD.

## Data Availability

Data in this study are available from the corresponding authors upon reasonable request.

## Supplementary Materials

Supplementary Materials Supplementary Table 1: specific primers used for RT-qPCR. Supplementary Table 2: FerrDEGs of IDD. Supplementary Table 3: classification of FerrDEGs. Supplementary Figure 1: three subnetworks of PPI and Metascape enrichment analysis. (a, b, c) Three subnetworks of PPI. (d, e, f) Relevant Metascape enrichment analysis of each subnetwork.
