# Supplementary material for: Establishment of Ferroptosis-Related Key Gene Signature and Its Validation in Compression-Induced Intervertebral Disc Degeneration Rats
Source: Oxid Med Cell Longev. 2023 Feb 10;2023:9020236. doi: 10.1155/2023/9020236 (PMC9937767; doi:10.1155/2023/9020236)
Supplement: Supplementary Materials — Supplementary Table 1: specific primers used for RT-qPCR. Supplementary Table 2: FerrDEGs of IDD. Supplementary Table 3: classification of FerrDEGs. Supplementary Figure 1: three subnetworks of PPI and Metascape enrichment analysis. (a, b, c) Three subnetworks of PPI. (d, e, f) Relevant Metascape enrichment analysis of each subnetwork. [file 9020236.f1.docx]

| Gene | Forward primer (5’->3’) | Reverse primer (5’->3’) |
| --- | --- | --- |
| AKR1C3 | CCCATCGTCCAGAGTTGGTC | TTGGCCAATCCTGCATCCTT |
| AR | CATCAAGCTGGAGAACCCGT | AGGCTAGCCAAGTCCCCATA |
| ATF3 | GACAGACAGCCCGCCTCTA | GACCTGGCCTGGATGTTGAA |
| EIF2S1 | CACTTCAGAATGCCGGGTCT | AACAAGCTGACATAGGCCCC |
| NQO1 | AGCGCTTGACACTACGATCC | TCTGCGTGGGCCAATACAAT |
| TXNIP | GATGCAAGGGTCTCAGCAGT | AGGCATGAACTGGAACTCGG |
| IL-1β | TTGAGTCTGCACAGTTCCCC | GTCCTGGGGAAGGCATTAGG |
| TGF-β1 | AGGAGACGGAATACAGGGCT | CCACGTAGTAGACGATGGGC |
| GAPDH | GCATCTTCTTGTGCAGTGCC | GATGGTGATGGGTTTCCCGT |

Supplementary Table 1: Specific primers used for RT-qPCR

Supplementary Table 2: FerrDEGs of IDD

| Gene symbol | log_2_FC | P.Value | Description | Regulation |
| --- | --- | --- | --- | --- |
| MUC1 | -0.62 | 0.00004 | mucin 1, cell surface associated | Down |
| ISCU | 0.50 | 0.00019 | iron-sulfur cluster assembly enzyme | Up |
| MMD | 0.90 | 0.00047 | monocyte to macrophage differentiation associated | Up |
| SLC2A12 | 1.02 | 0.00087 | solute carrier family 2 member 12 | Up |
| RRM2 | -0.31 | 0.00103 | ribonucleotide reductase regulatory subunit M2 | Down |
| MT1G | -1.68 | 0.00132 | metallothionein 1G | Down |
| GDF15 | 1.52 | 0.00158 | growth differentiation factor 15 | Up |
| PIR | 0.80 | 0.00242 | pirin | Up |
| ASNS | -0.42 | 0.00283 | asparagine synthetase (glutamine-hydrolyzing) | Down |
| AR | -0.28 | 0.00328 | androgen receptor | Down |
| SLC40A1 | 0.94 | 0.00353 | solute carrier family 40 member 1 | Up |
| ZNF419 | 0.18 | 0.00464 | zinc finger protein 419 | Up |
| ARRDC3 | 0.52 | 0.00672 | arrestin domain containing 3 | Up |
| RB1 | 0.40 | 0.00744 | RB transcriptional corepressor 1 | Up |
| AHCY | -0.66 | 0.00791 | adenosylhomocysteinase | Down |
| NQO1 | 1.52 | 0.00796 | NAD(P)H quinone dehydrogenase 1 | Up |
| NEDD4 | 0.60 | 0.00870 | NEDD4 E3 ubiquitin protein ligase | Up |
| AMN | 0.20 | 0.00872 | amnion associated transmembrane protein | Up |
| CEBPG | -0.43 | 0.01227 | CCAAT enhancer binding protein gamma | Down |
|  |  |  |  | (Continued) |
| (Continued) |  |  |  |  |
| Gene symbol | log_2_FC | P.Value | Description | Regulation |
| TXNIP | 0.78 | 0.01355 | thioredoxin interacting protein | Up |
| PEX10 | -0.31 | 0.01370 | peroxisomal biogenesis factor 10 | Down |
| MAP3K5 | 0.92 | 0.01486 | mitogen-activated protein kinase kinase kinase 5 | Up |
| ATM | 0.55 | 0.01497 | ATM serine/threonine kinase | Up |
| CAPG | -0.60 | 0.01552 | capping actin protein, gelsolin like | Down |
| EIF2S1 | -0.25 | 0.01842 | eukaryotic translation initiation factor 2 subunit alpha | Down |
| AKR1C3 | 1.58 | 0.01893 | aldo-keto reductase family 1 member C3 | Up |
| SLC2A1 | 0.44 | 0.02163 | solute carrier family 2 member 1 | Up |
| LAMP2 | 0.27 | 0.02300 | lysosomal associated membrane protein 2 | Up |
| EMC2 | 0.35 | 0.02314 | ER membrane protein complex subunit 2 | Up |
| OSBPL9 | 0.21 | 0.02436 | oxysterol binding protein like 9 | Up |
| PCK2 | -0.56 | 0.02682 | phosphoenolpyruvate carboxykinase 2, mitochondrial | Down |
| FTL | 0.14 | 0.03032 | ferritin light chain | Up |
| AIFM2 | 0.49 | 0.03269 | apoptosis inducing factor mitochondria associated 2 | Up |
| PANX2 | 0.20 | 0.03847 | pannexin 2 | Up |
| CARS1 | -0.30 | 0.04130 | cysteinyl-tRNA synthetase 1 | Down |
| CHMP5 | 0.32 | 0.04298 | charged multivesicular body protein 5 | Up |
| . |  |  |  | (Continued) |
| (Continued) |  |  |  |  |
| Gene symbol | log_2_FC | P.Value | Description | Regulation |
| BCAT2 | -0.35 | 0.04367 | branched chain amino acid transaminase 2 | Down |
| GPX4 | 0.45 | 0.04559 | glutathione peroxidase 4 | Up |
| GABPB1 | -0.44 | 0.04601 | GA binding protein transcription factor subunit beta 1 | Down |
| ATF3 | -0.54 | 0.04814 | activating transcription factor 3 | Down |

| Driver | Suppressor | Marker |
| --- | --- | --- |
| MMD, AMN, PEX10, ATM, EMC2, OSBPL9, CARS1, ATF3 | MUC1, ISCU, RRM2, MT1G, GDF15, PIR, AR, SLC40A1, RB1, AHCY, NQO1, NEDD4, AKR1C3, LAMP2, AIFM2, PANX2, CHMP5, BCAT2, GPX4 | SLC2A12, RRM2, GDF15, ASNS, ZNF419, ARRDC3, CEBPG, TXNIP, MAP3K5, CAPG, EIF2S1, SLC2A1, PCK2, FTL, GPX4, GABPB1, ATF3 |

Supplementary Table 3: Classification of FerrDEGs


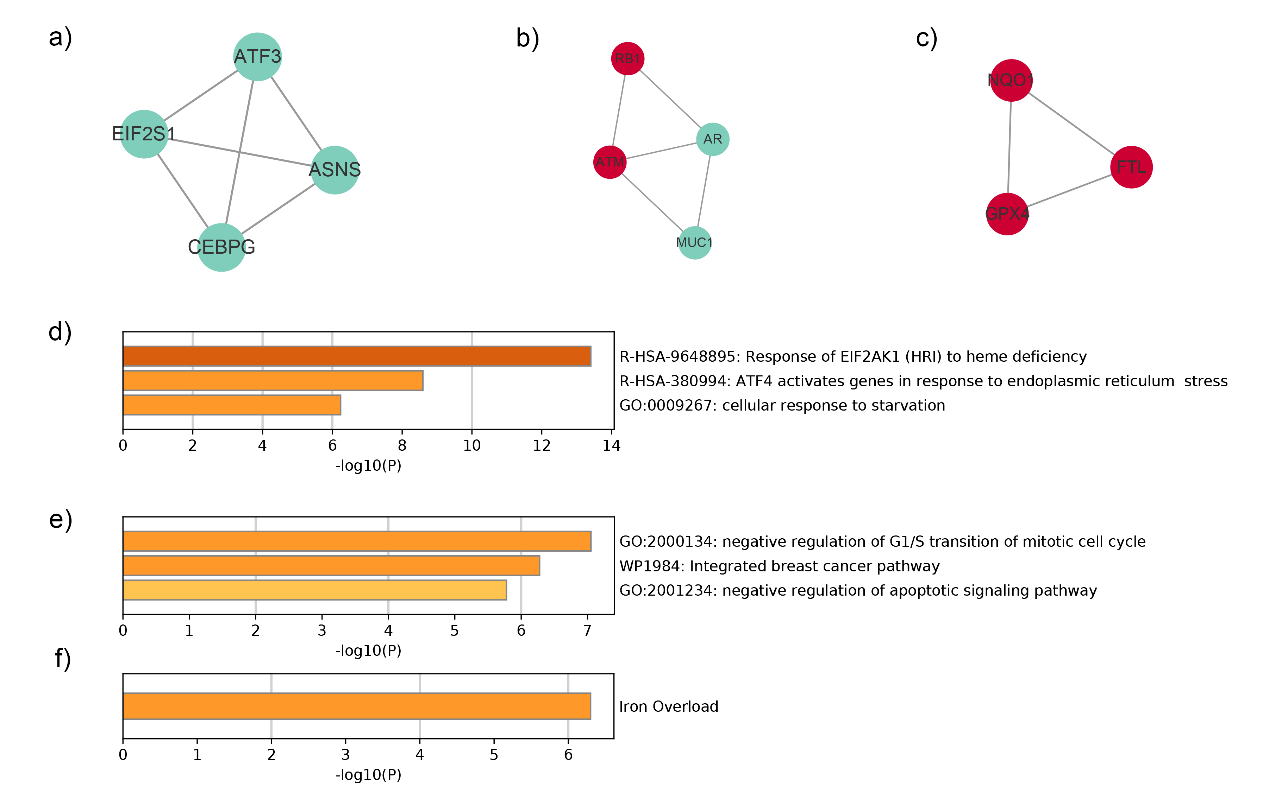


Supplementary Figure 1: Three subnetworks of PPI and Metascape enrichment analysis. (a, b, c) Three subnetworks of PPI. (d, e, f) Relevant Metascape enrichment analysis of each subnetwork.
